# Impact of implant location on load distribution of implant-assisted removable partial dentures: a review of in vitro model and finite-element analysis studies

**DOI:** 10.1186/s40729-023-00500-3

**Published:** 2023-09-19

**Authors:** Hiromi Ichikawa, Nobuhiro Yoda, Toru Ogawa, Maya Iwamoto, Tetsuo Kawata, Hiroshi Egusa, Keiichi Sasaki

**Affiliations:** 1 Division of Advanced Prosthetic Dentistry, Tohoku University Graduate School of Dentistry, 4-1, Seiryo-Machi, Sendai, Miyagi 980-8575 Japan; 2 Dental Technical Training School attached to Nihon University School of Dentistry, Tokyo, Japan; 3 Department of Prosthodontics, Faculty of Dental Medicine, Universitas Airlangga, Surabaya, 60132 Indonesia; 4 Division of Molecular and Regenerative Prosthodontics, Tohoku University Graduate School of Dentistry, Sendai, Miyagi 980-8575 Japan

**Keywords:** Dental implant, Implant-assisted removable partial denture, In vitro model, Finite-element analysis, Load distribution

## Abstract

**Background:**

Appropriate load distribution among the supporting elements is essential for the long-term success of implant-assisted removable partial dentures; however, there is little information available on load distribution.

**Purpose:**

This study aimed to evaluate the effect of implant location on load distribution in implant-assisted removable partial dentures by reviewing in vitro models and finite-element analysis studies.

**Materials and methods:**

English-language studies which examined the load distribution of implant-assisted removable partial dentures and were published between January 2001 and October 2022 were extracted from PubMed, ScienceDirect, and Scopus online databases, and manual searching. Two reviewers selected the articles based on the predetermined inclusion and exclusion criteria, followed by data extraction and analysis.

**Results:**

Forty-seven studies were selected after evaluating the titles and abstracts of 264 articles; two were identified manually. After screening the text, 12 studies were included: six in vitro model experiments and six finite-element analysis studies. All included studies used a mandibular free-end missing model (Kennedy Class I or II). The influence of implant location on load distribution to the abutment tooth, implant, and mucosa under the denture base was summarized in three cases: implant at the premolar, first molar, and second molar region. Due to differences in the measurement method of load distribution and loading condition to the denture, the results differed among the studies.

**Conclusions:**

The implant location in implant-assisted removable partial dentures can affect load distribution to the supporting elements, such as the abutment tooth, implant, and mucosa under the denture base.

**Supplementary Information:**

The online version contains supplementary material available at 10.1186/s40729-023-00500-3.

## Background

Recently, the effectiveness of implant-assisted removable partial dentures (IARPDs), in which a few implants are placed under the base of removable partial dentures, has been demonstrated [1–5]. This type of removable partial denture is referred to as implant-supported RPD or implant-retained RPD, depending on the role of the implant. IARPD aims to prevent the rotation and subsidence of RPD’s extension base and improve denture stability by the implant placed in the distal part of the mandibular free-end missing. The basic strategy of adding an implant support element to the free-end missing is to enable the defect type to transform into a pseudo-intermediate defect (pseudo-Kennedy class III) [6–8].

The occlusal force increases in IARPD wearers, and it is expected to recover oral function better than conventional RPD (CRPD) [9]. Furthermore, IARPD improves patient satisfaction and nutritional intake with enhanced masticatory function [10–12]. However, complications, such as loosening of the attachment and abutment screws on the implant or fracture of the denture base and framework, need to be noted [5, 7, 12–16]. Therefore, establishing appropriate guidelines regarding the IARPD design warrants a suitable selection criterion, including the number, location, and size of implants. However, there is a high degree of freedom in the design and wide variation in the IARPD clinical conditions, which makes it difficult to perform high-quality clinical comparisons among various IARPD designs.

One mechanical feature of the IARPD involves the complexity of the supporting elements against the occlusal force on the denture [7, 13]. The occlusal force applied to the denture during function gets transmitted to three supporting elements with different amounts of deviation against pressure: the abutment tooth, mucosa under the denture base, and implant. This necessitates considering the appropriate load distribution to the supporting elements and understanding the load-bearing aspect of each support element. However, simultaneous measurement of these loads during function in the human oral cavity is difficult because of several barriers, such as the lack of a suitable measuring device with proper size and accuracy, and difficulties in securing the participants [17].

Studies using in vitro model experiments or finite-element analysis (FEA) have investigated the load distribution of IARPD owing to the above-mentioned limitations of clinical comparison. The findings of these simulation studies are useful for determining the effect of clinically selected factors, such as implant placement or its location and the type of attachment, on the load distribution in IARPD. Conversely, there is still no consensus regarding the load distribution, because the experimental studies were performed under various estimates and assumptions. Moreover, the experimental conditions differed across studies. Therefore, we aimed to summarize and review the literature with experimental studies, including in vitro model experiments and FEA conducted on the load distribution to the supporting elements of IARPD, and examine the effect of implant location on the load distribution as one of the essential factors of IARPD design.

## Methods

### Literature search strategy

An electronic search was performed using MEDLINE (via PubMed), Science Direct, and Scopus as the database research tools. The keywords used for the research were general: ((((((in vitro) OR (model)) OR (mechanics)) OR (computer simulation)) OR (computational)) OR (finite-element)) AND (((Implant-retained removable partial denture) OR (Implant-supported removable partial denture)) OR (Implant-assisted removable partial denture)) to allow the extraction of relevant data. Moreover, we performed a manual search by examining the bibliography of the identified articles for potentially relevant studies.

### Inclusion and exclusion criteria

The inclusion criteria were as follows: complete manuscripts that reported the effect of implant location on the load distribution to the supporting elements of IARPD, such as the mucosa, abutment tooth, and implant, using in vitro model experiments or FEA. IARPD for three or more teeth-free-end missing was targeted. Only articles published in English from January 2001 to October 2022 were included in this study.

The exclusion criteria were: reviews, in vivo clinical studies, animal studies, no dental application, and no quantitative stress and load measures on the supporting elements of IARPD. All selected articles were collected, of which the required data were extracted, and duplicate articles were excluded.

### Study selection

**Fig. 1:**
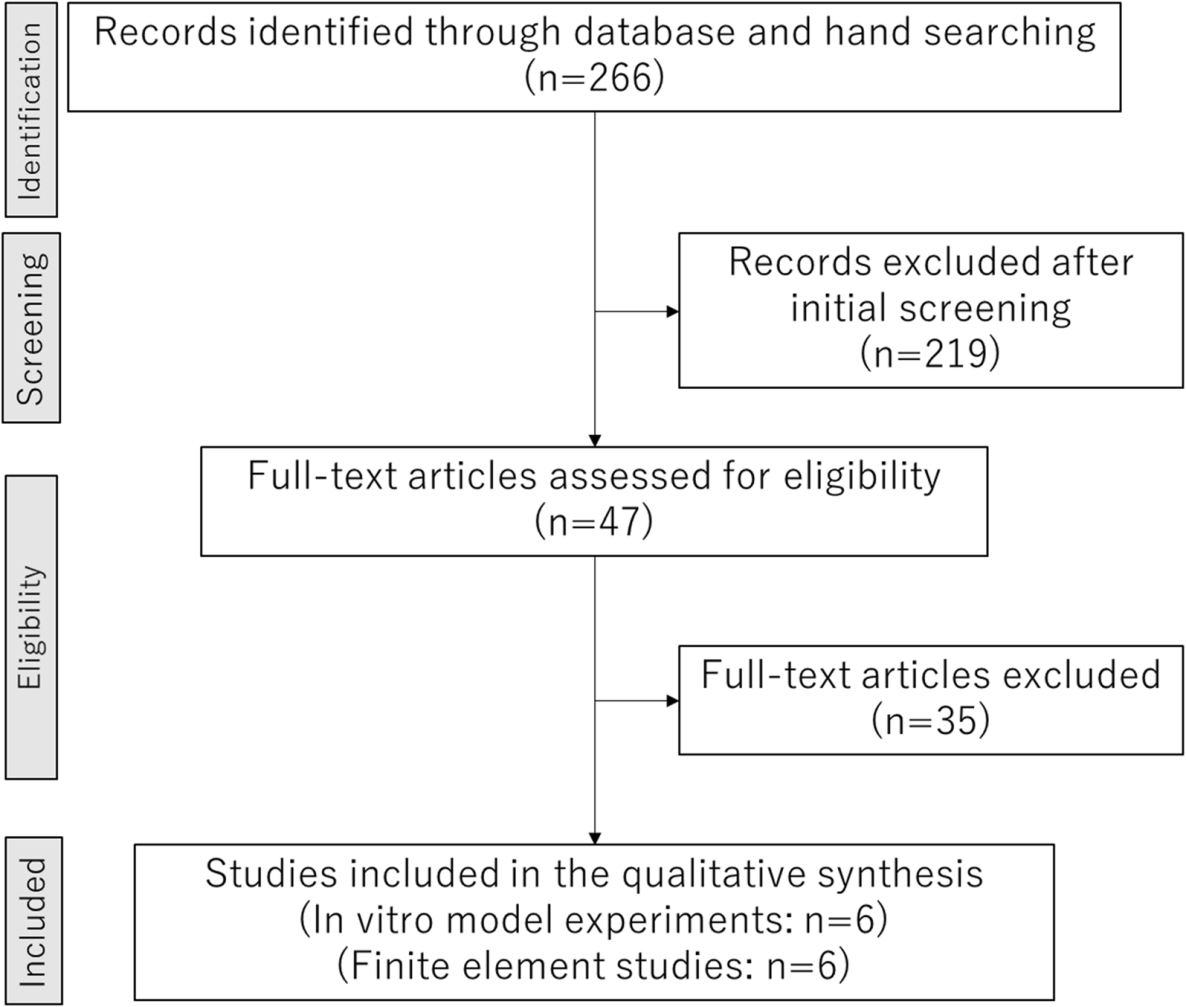
Study selection flow chart

Figure 1 illustrates the strategy used for the literature search. The first two authors performed the initial search (HI and NY) and screened the titles and abstracts of the data sources for approximately 1 month. Upon identifying an article relevant to the study’s objective, its references were manually screened to identify additional studies that met the inclusion criteria. Second, the complete texts of these articles were read to examine the details of the reported results. Subsequently, the reviewers (TO and MI) confirmed the concurrence of the results, and discrepancies between the results of the two authors were discussed. Eventually, we included studies that investigated the effect of implant placement and its influence on the load distribution to the supporting elements of the IARPD using in vitro model experiments or FEA.

### Data collection and items

An extraction sheet was created for data collection using Microsoft Excel (Microsoft Office Professional 2019, WA, USA). The table for the in vitro model study contained the following information: author, publication year, model information (missing teeth area, Kennedy classification, and materials), denture design, implant information (number, location, and attachment), loading condition, sensors for measurement, and results. Similarly, the table for the FEA study contained the following information: author, publication year, FE model information (missing teeth area, 2 dimensional (2-D) or 3-D, Kennedy classification, and material properties), denture design, implant information (number, location, and attachment), loading condition, measured stimulation, and results. A literature review was performed after summarizing the results for each subfield.

## Results

### Search results

The initial search yielded 264 citations published between 2001 and 2022. Two hundred nineteen articles were irrelevant to the topic based on the titles and abstracts; thus, they were excluded, resulting in 45 articles for additional search. We identified two articles by hand-search based on the bibliography. The full texts of 47 articles were thus assessed to determine those that investigated the effect of implant location on the load or stress distribution to the supporting elements in IARPD. Eventually, 12 studies met the inclusion criteria (Fig. 1): six articles used in vitro model experiments, and six used FEA.

Studies not included (*n* = 35) in the review after reading the full texts and reasons for exclusion are listed in Additional file 1: Table S1.

### Experimental condition of the in vitro model studies

Table 1 summarizes the six selected articles [17–22]. All studies employed a mandibular free-end missing model (Kennedy Class I or II) in an improved ready-made resin model. A pseudo-mucosa of 2 mm thickness made of silicone impression material was installed on the residual ridge area in all studies. In most studies, a pseudo-periodontal ligament (PDL) comprising of silicone impression material was installed around the abutment tooth root. The RPD design varied across studies. RPA clasps (rest, proximal plate, and Akers clasps) [21, 22], RPI clasps (rest, proximal plate, and I-bar clasp) [17, 18], Akers clasps, and clasp-less type (rest and bracing arms only) [20], or other types of retainers [18, 19] were used as the direct retainers. In terms of the abutment on the implant, one study used a healing abutment predominantly as support [20], whereas others used an attachment-type abutment [17–19, 21, 22]. For the loading condition, a static load was vertically applied to the occlusal surface of the IARPD at a constant crosshead speed. Still, the magnitude of the load and loading point varied across studies. Some studies employed unilateral and bilateral loading [18, 21], whereas others utilized only unilateral loading [17, 19, 20, 22]. Kihara investigated the effect of the loading location [20]. Strain gauges [18–22] and piezoelectric transducers [17] were used to measure the load to the tooth, implant, and surrounding tissues or residual ridge. A seat-type sensor was used to measure the load on the mucosa under the denture base [17].

**Table 1:** Studies comprising in vitro model experiments

Study	Experimental model					Denture design		Implant		
	Missing area	Kennedy classification	Materials of model	Artificial PDL	Artificial mucosa	Retainer for direct abutment tooth	Framework (major connector)	System	Location	Abutment (attachment)
Hegazy [18]	34, 35, 36, 37 and 44, 45, 46, 47	Class 1	Acrylic resin	Silicone impression material	2-mm-thick silicone layer	Group 1: Claspless sub-group A: horizontal bracing arms sub-group B: vertical bracing arms Group 2: an RPI clasp.	Cobalt–chromium alloy metal frame	Helix ART, Dyna Dental Engineering	Group 1: 34, 44 Group 2: 36, 46	Ball-abutment
Matsudate [17]	45, 46, and 47	Class 2	Epoxy resin model (D50–520; Nissin, Kyoto, Japan)	Approximately 0.5-mm-thick, silicone impression material	Approximately 2-mm-thick silicone impression material	RPI clasp	Lingual plate, double Akers clasp for the left second premolar and first molar	Standard RN	45 or 47	Ball attachment
ELsyad [19]	(Two patterns) 35, 36, and 37 and 45, 46, and 47 or 34, 35, 36, and 37 and 44, 45, 46, and 47	Class 1	Acrylic resin model	Not described	2-mm-thick self-cure silicon layer	No clasps were used (rest only)	Lingual bar	Laboratory implants (TioLogic; Dentaurum, Ispringen, Germany): 3.7 × 13 mm	Group 1: 34 and 44 or 37 and 47 Group 2: 35 and 45 or 37 and 47	Ball-socket attachments
Kihara [20]	45, 46, and 47	Class 2	Acrylic resin model (E50–520, NISSIN, Kyoto, Japan)	1.0-mm-thick polyvinyl siloxane impression material	2-mm-thick polyvinyl siloxane impression material	Akers clasp	Lingual plate, a double Akers clasp for the left second premolar and first molar	Zimmer Biomet Dental, Palm Beach Gardens, FL, USA: 3.75 × 10 mm	46 and 47	A temporary healing abutment with a 4.0 mm height
Rungsiyakull [21]	35, 36, and 37 and 45, 46, and 47	Class 1	Acrylic resin model	Silicone impression material	2-mm-thick silicone layer	Rest-Proximal plate–Akers clasps	Lingual bar	Mini-implants (PW+, Nakhon Pathom, Thailand): 2.75 × 10 mm	35 and 45, or 36 and 46 or, 37 and 47	Equator attachments
Naing [22]	34, 35, 36, 37, and 44, 45, 46, 47	Class 1	Acrylic resin (Nissin E1–550)	Not described	2-mm-thick silicone layer (Exahiflex; GC)	Rest-proximal plate–Akers clasps	A lingual bar and a free-end saddles	BL tapered SLA, Straumann (10 × 4.1 mm)	34 and 44, or 37 and 47	Locator attachments and Magnetic attachments

Study	Loading condition magnitude/direction/speed/location	Sensors manufacture/location	Findings related to the effect of the implant or its position on the load distribution or denture displacement
Hegazy [18]	70 N, vertical, four points bilaterally or two points unilaterally (second premolar and first molar)	Eight self-protected linear strain gauges (Tokyo Sokki Kenkyujo) were cemented onto the buccal and lingual surfaces of the abutment teeth and implants	Stress transmitted to the abutment tooth and implant in the distal implant IARPD was significantly smaller than those of mesial implant IARPD
Matsudate [17]	100 N, vertical, one point (right M1 on denture), unilateral	Piezoelectric 3D force transducers (Kistler Instruments AG, Winterthur, Switzerland) were used to measure the tooth and implant load A pressure-sensitive tactile sensor film (I-SCAN; Nitta, Osaka, Japan) was used to measure the pressure on the mucosa	The load on the abutment tooth was larger with distal implant-supported RPD than with CRPD Distal implant-supported RPD greatly reduced the load beneath the denture base The lateral component of the load that was exerted on the abutment tooth and the implant was larger with mesial implant-supported RPD than with distal implant-supported RPD
ELsyad [19]	60 N, vertical, one point (right M1 on denture), unilateral	Three linear strain gauges (Kyowa Electronic Instruments Co, Ltd, Tokyo, Japan) were cemented at each implant’s buccal, lingual, and distal surfaces at the loading and non-loading sides	The distal implant position showed significantly higher peri-implant stresses than the mesial implant position
Kihara [20]	100 N, vertical, comparing three points (#45, #46 and #47), unilateral	Four strain gauges (KYOWA electric-corporation, Tokyo, Japan) were attached to the implant and tooth root surface	Bending moments of the abutment tooth and implant were significantly higher in mesial implant than in distal implant-supported PRD The largest mesio-distal displacement of the abutment tooth was observed in mesial implant-supported RPD under #47 loading. In mesial implant-supported RPD, a higher bending moment of the abutment tooth under #45 and #47 loading was detected, although the bending moment in distal implant-supported RPD was almost zero Bending moments in the implant in mesial implant-supported RPD were statistically larger than distal implant-supported RPD under all loading conditions
Rungsiyakull [21]	150 or 200 N, vertical, four points bilaterally or two points unilaterally (second premolar and first molar)	Twelve strain gauges (Kyowa Electronic Instruments Co, Ltd, Tokyo, Japan) were bonded on the mesial and distal surfaces of the model adjacent to the first thread region of each dental mini-implant, 1 mm away from the implant body and perpendicular to the occlusal plane Two strain gauges were bonded on the buccal surface and parallel to the long axis of the primary abutment tooth	A mesially placed implant decreased microstrains around abutment teeth compared to a distally placed dental mini-implant A distally placed implant decreased microstrains around the dental mini-implant itself
Naing [22]	120 N, vertical, one point (right first molar on denture), unilateral	The fine lead wires of the strain gauges (Kyowa Electronic Instruments, Tokyo, Japan) were attached to the flattened and smoothed surface of the experimental model at the mesial, distal, buccal, and lingual sides of each implant	On the loading sides, a larger strain around the implant was observed in the implant at the molar area than in the premolar area The maximum principal strain (MPS) was distolingually distributed on the loading side under all experimental conditions. On the non-loading side, the MPS distribution of the molar IARPD with the locator attachment was in the distolingual direction. In contrast, the MPS distributions of the other experimental conditions were mesiolingually distributed

*PDL* periodontal ligament, *RPI* rest, proximal plate, I bar, *RN* regular neck, *IARPD* implant-assisted removable partial denture, *CRPD* conventional removable partial denture design

### Experimental condition of the FEA studies

Table 2 summarizes the selected six studies [23–28]. All studies employed mandibular free-end missing models similar to the in vitro model experiments. The studies comprised a 2-D [23] and 3-D FEA [24–28], although most performed the analysis in the partial jawbone. In recent years, for 3-D FEA, researchers have adopted more realistic models, such as those based on human computed tomography (CT) images [25, 26, 28] or scanned skull models [27]. Most studies set up the material properties of bone, teeth, and PDL as homogeneous and isotropic linearly elastic. However, one study used the heterogeneous material property for the PDL [26]. In terms of the direct retainer in RPD, an RPA clasp [25, 28], Akers clasp [26], and RPI clasp [27] were used. Contrarily, the retainer arms were not used in other studies [23, 24]. The implant location settings also varied across studies. These studies used several types of abutments, such as healing abutments [23, 24, 27], telescope crowns [25], and specific attachment systems [26, 28]. In addition, they applied various loading conditions; unlike in the model experiment, some studies reproduced the oblique directional or horizontal directional load and vertical directional load on the IARPD [25, 26]. Most studies applied multi-point loading, and one study defined the loading condition using a contraction vector of the masticatory muscle activities, thus reproducing a more realistic situation [27].

**Table 2:** Studies of the finite-element analysis

Study	Finite-element model of the jaw bone				Denture design		Implant		
	Missing tooth	2-D or 3-D (model)	Kennedy classification	Material properties	Clasp for direct abutment tooth	Framework	Location	Number (functioned)	Abutment (attachment)
Cunha [23]	34, 35, 36, and 37	2-D	Class 1 (partially edentulous Hemi-arches)	Homogeneous, isotropic, and linearly elastic, except for the PDL, which was considered a non-homogeneous structure	Not described Only rest (from the illustration)	Metal framework	Three patterns: 35, 36 or 37	1	Healing abutment
Memari [24]	35, 36, 37 and 45, 46, 47	3-D	Class 1	All living tissues were presumed elastic, homogeneous, and isotropic	Not described Only rest (from the illustration)	Metal framework	Three patterns: 35, 45 or 36, 46, or 37, 47	2	Not described Healing abutment (from the illustration)
Xiao [25]	34, 35, 36, 37 and 44, 45, 46, 47	3-D (CT-based patient-specific model)	Class 1	All materials were presumed to be linearly elastic, homogeneous, and isotropic	Classic RPA clasps	Metal framework	Three patterns: 45, 46, or 47	1	Rigid telescopic crown
Ortiz-Puigpelat [26]	34, 35, 36, 37 and 44, 45, 46, 47	3-D (CT-based patient-specific model)	Class 1	Isotropic linear for all elements of the model, except for the PDL of the canine, which was established as non-linear in geometry	Akers clasp	Metal framework	Four patterns: 35, 36, or 37 or control (without implant)	1	Locator attachment
Ohyama [27]	35, 36, and 37	3-D [a skull replicative model (P10–SB1, Nissin, Kyoto, Japan)]	Class 2	Isotropic structural non-linear static analysis	RPI clasp	A mesial rest for the right primary premolar, an Akers clasp for the right first molar, and a lingual bar	Three patterns: 35, 36, or 37	1	Two patterns of healing abutment: mucosal-level (2-mm-height)
Jia-Mahasap [28]	35, 36, 37 and 45, 46, 47	3D (based on the scanned data of the mandibular model, RPD framework, and mini dental implant)	Class 1 (entire mandible)	Linearly elastic, homogenous, and isotropic	RPA clasp	Cobalt–chrome–molybdenum framework, with lingual bar	Three patterns: 35, 36, or 3716.5 mm, respectively	1	Equator attachment

Study	Loading condition magnitude/direction/speed/location	Measured stimulation	Findings [related to the effect of the implant or its position on the load distribution (or denture displacement)]
Cunha [23]	Multi-point loading (on abutment tooth and denture): vertical force of 50 N on each cuspid point and 400 N in the models with RPD	The von Mises equivalent stress	The presence of an implant reduced the denture displacements Stress in the PDL and spongy bone was the largest in CRPD, followed by implant at P2, M1, and M2 second molar Stress in cortical bone was the largest in CRPD, followed by implant at M2, M1, and P2 The stress in fibromucosa was the largest in CRPD, followed by the implant at P2, M2, and M1
Memari [24]	Multi-point loading (on abutment tooth and denture): loading was 10 N at each tooth location (on the second molar, the first molar, the second premolar, and the first premolar) in the vertical direction. (total load of 80 N at both sides)	Displacement and von Mises stress	Stress on the abutment tooth was the largest in the implant at P2, followed by M1 and M2 The largest stress was observed in the implant at M2 Cortical bone stress and implant stress were the largest in the implant at P2, followed by M2 and M1 Spongy bone stress was the largest in the implant at M2, followed by M1 and P2
Xiao [25]	Multi-point loading (on denture): three different static load scenarios on the center point of each simulated artificial tooth: 100 N in the vertical direction, 100 N at a 45° inclination buccolingual direction, 20 N in the horizontal buccolingual direction	The maximum EQV stress value The maximum mucosa displacements	Vertical loading condition: stress on the PDL of the abutment tooth was the largest in CRPD, followed by implant at M2, P2, and M1. Stress on mucosa under the denture base was largest in CRPD, followed by P2 and M2 (similar), and M1. In cortical and spongy bones, stress was the largest in the implant at P2, followed by M2 and M1 Oblique loading condition: stresses on the PDL of the abutment tooth, mucosa under the denture base, and bones were the largest in CRPD, followed by implant at M2, P2, and M1 Horizontal loading condition: stress on the PDL of the abutment tooth was the largest in CRPD, followed by implant at M2, P2, and M1. Stress on mucosa under the denture base and cortical bone was largest in CRPD, followed by P2, M2, and M1 (similar). Stress in spongy bone was the largest in the implant at P2, followed by M1 and M1
Ortiz-Puigpelat [26]	Multi-point loading: (on tooth and denture) Vertical force of 200 N directed towards occlusal surface The oblique inclination of a 30° angle in relation to the occlusal plane	Denture displacement von Mises stress maps in MPa, non-linear strains in % of the different structures	Mandible stress was the largest in CRPD, followed by implant at M2, M1, and P2 Soft tissue stress was the largest in the implant at P2, followed by M1, CRPD, and M2 Implant stress was the largest in the implant at P2, followed by M2 and M1 PDL (abutment tooth) stress was similar in all conditions, but PDL deformation was the largest in CRPD, followed by implant at M1, M2, and P2 (M2 and P2 are similar) Abutment tooth stress was largest in CRPD, followed by implant at M1, M2, and P2 (M2 and P2 are similar)
Ohyama [27]	Loading of the mandible by masticatory muscles during biting in the intercuspal position	The displacement of the abutment tooth (P1) and denture base Minimum principal stress of the cortical bone around the implant neck	The denture base and tooth displacement at an abutment height of 0 mm was larger than 2 mm In abutment height of 0 mm, displacement of abutment tooth in the implant at P2 was largest, followed by M1 and M2. In abutment height of 2 mm, displacement of abutment tooth in the implant at M2 was largest, followed by M1 and P2 In abutment height of 0 mm, minimum principal stress in the implant at M2 was the largest, followed by M1 and P2. In abutment height of 2 mm, minimum principal stress in the implant at P2 was the largest, followed by M1 and M2
Jia-Mahasap [28]	Two-point loading (on denture): the vertical load of 100 N was applied bilaterally on the denture base at 9 and 14 mm distal from the first premolar abutment tooth (50 N each)	The volume average of von Mises stress was calculated on the abutment tooth, mini dental implant, and surrounding bone	Stress in the abutment tooth was the largest in the implant at P2, followed by M1 and M2 The stress of the implant was largest in the implant at M1, followed by P2 and M2 Stress in the bone was largest in the implant at M1, followed by M2 and P2

*RPI* rest, proximal plate, I bar, *RPA* rest, proximal plate, Akers, *EQV* equivalent stress (maximum von Mises stress), *PDL* periodontal ligament, *P2* second premolar region, *M1* first molar region, *M2* second molar region

### The effect of the implant location on the load distribution

Implant location was classified into three patterns, namely, the premolar region (near the abutment tooth), the first molar region, and the second molar region, for the convenience of summarizing all the included studies. For summarizing the load distribution, the load on the abutment tooth, including the mechanical stimulation in its PDL, mucosa under the denture base, and implant as supporting elements in the IARPD, were considered, respectively.

In model experiments, Matsudate et al. demonstrated that the total load on the abutment tooth was most prominent in the case of an implant at the second molar region [17]. Similar results were obtained in a study that measured the stress in the bone around the abutment tooth [21]. On the other hand, the lateral component of the load [17] or the bending moment [18, 20] on the abutment tooth was larger in the implant at the premolar region. Regarding the load on the mucosa, the IARPD with the implant at the second molar significantly reduced the load under the denture base compared to CRPD [17]. In terms of load on the implant, some studies demonstrated that the distal implant position showed significantly higher load on the implant [17] or peri-implant stresses [19, 22] than that for the mesial implant position, whereas others showed the opposite results [18, 20, 21].

In FEA studies, the stress in the PDL or movement of the abutment tooth was analyzed in addition to the load on the abutment tooth itself. Memari et al. and Jia-Mahasap et al. reported that the stress on the abutment tooth was largest in the implant location at the second premolar, followed by the first and second molar [24, 28]. In addition, Cunha et al. showed that the stress in the PDL was largest in CRPD, followed by implant location at the second premolar, first molar, and second molar [23]. Contrarily, Xiao et al. demonstrated that stress in PDL of the abutment tooth was largest in CRPD, followed by implant location at the second molar, second premolar, and first molar [25]. Another study showed no significant influence of the implant location on stress in PDL [26]. In addition, Ohyama et al. showed that in the case of implant abutment height of 0 mm, displacement of the abutment tooth was the largest in implant location at the second premolar, followed by the first and second molar. The order was reversed in the case of implant abutment height of 2 mm [27].

Regarding the load on mucosa under the denture base, Cunha et al. showed that the stress in the fibromucosa was largest in CRPD, followed by implant location at the second premolar, second molar, and first molar [23]. Xiao et al. demonstrated the smallest stress on the mucosa at the implant location at the first molar irrespective of the loading direction [25]. On the other hand, Ortiz-Puigpelat et al. demonstrated that the largest soft tissue stress was observed in the implant location at the second premolar, followed by the first and second molar. However, there were no significant differences between molars [26]. In terms of the load on the implant, the minimum principal stress in the implant at the second molar region was the largest, followed by the first molar and second premolar region with an abutment height of 0 mm, and the order was reversed with an abutment height of 2 mm [27].

Most FEA studies demonstrated the stress in whole mandibular bone and did not focus on the peri-implant or peri-abutment tooth area. Ortiz-Puigpelat et al. mentioned that the implant at the first molar region offered a more favorable distribution and dissipation of stress along the entire length of the peri-implant bone [26]. On the other hand, another study showed that implant stress was most extensive in the implant at the first molar, followed by the second premolar and second molar regions [28].

## Discussion

Considering the difficulty of in vivo investigation, simulation studies are valuable in investigating the load distribution of IARPD, although the number of studies is limited. This review summarized the current biomechanical findings regarding the load distribution of IARPD from in vitro model experiments and FEA studies. These studies included various biomechanical aspects of IARPD; however, the review focused only on implant location’s effects on load distribution in the mandibular free-end missing.

It was difficult to determine the better method for elucidating the biomechanics of IARPD, considering the advantages and disadvantages of each study design. Simulation studies should ideally use models reproducing the details of human jawbone morphology or properties and applying the real loading conditions. For example, 3-D FEA is generally superior to 2-D FEA. FEA can be more effective in investigating the stress/strain distribution in the jaw bone. However, in vitro model experiments might be able to make more sense of denture behavior. In most FEA studies, the clasp on the abutment tooth completely adhered to the tooth, which is not observed in the clinical situation. Understanding the characteristics of each simulation study before interpreting the results is essential.

In the in vitro model studies, it is challenging to imitate living tissues, such as the jawbone, mucosa, and teeth. Although researchers used an artificial mucous membrane and PDL using silicon materials in the model studies, the thickness and elasticity substantially vary among individuals in vivo. Similar to the model studies, it is unclear if the material properties of jawbones, PDL, and other components used in FEA studies were biologically relevant. Recent FEA studies have generalized nonlinear heterogeneous material properties based on a specific patient’s CT data, enabling more realistic simulation research [29, 30].

For the denture design of the IARPD, all studies used metallic frames. Previous FEA studies have demonstrated that the occlusal rest position or attachment system affects the strain on the metallic frame of the IARPD [31, 32]. Nogawa et al. [33] also compared the biomechanical behavior of three types of direct retainers of IARPD; however, further studies are still required to consider the retainers’ effect on load distribution. Elsyad et al. [19] compared the number of free-end missing teeth and clarified that the long saddle of IARPD recorded significantly higher peri-implant stresses than the short saddle. Further studies are needed to clarify the effect of the number of missing teeth on IARPD behavior.

In terms of loading conditions, a static load was applied to the IARPD in both in vitro and FEA studies. The magnitude of the applied load ranged between 50 and 200 N, thus simulating an occlusal force during clenching or chewing. Although only vertical load was applied in the model studies, oblique or horizontal direction loads were additionally applied to the FEA. However, in clinical scenarios, various directional dynamic loads are exerted on the tooth and implant during chewing [34–36]. The model study applying dynamic and static loading conditions to the denture demonstrated significant differences in the load distribution between loading conditions in the mandibular implant-supported overdenture [37]; therefore, the dynamic loading condition should be included in the simulation studies.

To understand the load distribution of the IARPD, the loads applied to each supporting element (abutment tooth, implant, and mucosa under the denture base) of the IARPD should be ideally measured three-dimensionally, simultaneously, and accurately. Strain gauges and piezoelectric transducers were mostly used to measure the load or stress of the supporting elements in model studies. In the included studies, the strain gauges were attached directly to the implant body [18–20] or the resin part around the implant [21, 22]. Considering the load in the peri-implant bone, the latter might be more meaningful, because the distortion of the surrounding bone can be more related to bone damage or remodeling. The piezoelectric transducer method can effectively and accurately measure the 3-D load on the implants and abutment tooth because of its favorable characteristic of load measurement in vivo [38]. For measuring the load under the denture base, Matsudate et al. used seat-type sensors [17], which were also used in vivo previously [39, 40]. However, a thin seat-type sensor with a larger sensing area may be ideal for understanding the load distribution in this area. Alternatively, FEA studies can evaluate the magnitude and distribution of the stress/strain in the bone, mucosa, and PDL. Although there are no explicit guidelines regarding the kind of stresses that should be used in the FEA for dental biomechanics, principal stresses and von Mises stresses are often used equally. Since minimum principal stress represents the peak compressive stress, the evaluation of that stress value could provide valuable information for understanding bone remodeling [41]. In the FEA studies included in this review, Ohyama et al. used the minimum principal stress for evaluating the distribution of mechanical stimulation in the model [27]. Since the accuracy and clinical validity of the FEA results are highly dependent on the reproducibility and condition settings of the model, more recent studies may be generally reliable due to the development of computational technologies. On the other hand, the FEA studies in this review have not been verified using clinical outcomes. Therefore, although the usefulness of FEA is understood, the clinical validity of such simulation results might not be high. This means that clinical validity must be carefully considered when interpreting FEA results, even in model experiments.

Regarding load distribution, the loads applied on the abutment tooth, the implant, the mucosa under the denture base, and their balance were considered. With regard to the load on the abutment tooth, the load magnitude and direction, as well as the stress on the PDL and the surrounding bone should be considered. Model studies revealed that the load on the abutment tooth increased when implants were placed in the second molar region, and the bending moment became larger when implants were placed in the premolar region. In particular, Matusdate et al. demonstrated a larger load on the abutment tooth in the IARPD with the implant location at the second molar compared with CRPD [17], which means that the implant placement does not necessarily reduce the burden on the abutment tooth in IARPD. When focusing on the stress in PDL or bone around the abutment tooth, the stress can be larger in the implant location at the second molar region than in other regions from the model experiment results or FEA [25, 27].

On the contrary, some studies showed that placing the implant closer to the abutment tooth caused more strain on the abutment tooth [23, 24, 28]. However, considering the contour diagrams of FEA results, the higher stress area was larger in the implant at the second molar region than in other regions in the above studies [23, 28]. This can be explained by the fact that the denture can rotate on the implant as a fulcrum, which may reduce the load’s vertical components but increase the load’s lateral components on the abutment tooth [17], causing more strain on the abutment tooth. Denture rotational movement can also be affected by the loading condition, namely, whether the loading point on the denture is anterior or posterior to the implant location [20]. In addition, Ohyama et al. suggested that denture and abutment tooth movement can be controlled by the bracing effect of the implant abutment [27]. If the denture behavior can be controlled well by the implants placed at the premolar area, the burden on the remaining teeth may reduce, protecting the remaining teeth. The survival rates of abutment teeth used to retain and/or support the IARPD were reported to range from 79.2 to 100% [42], which might be better than that (73.6%) of the abutment tooth in conventional RPD [43]. The implant support and/or retention in IARPD can avoid the swing movements along the axis of rotation of the prosthesis, which may reduce the risk of abutment tooth loss. Appropriate oral hygiene and a regular control and maintenance program are also essential to reduce the risk of failure of abutment teeth [42].

Considering the overall stress distribution in the mucosa area, the implant at the first molar area may minimize the total stress in that area [23–25]. Some studies showed that placing the implant under the denture base reduced the load on the mucosa [17, 28]. The previous model experiments [44, 45] also demonstrated minimized mucosal pressure upon placing the implant in the second molar area. It is to be noted that, as described above, even if the implant location was the same, the load under the denture base can change depending on the loading points on the denture [20]. When the loading point is set between the implant and the abutment tooth, the load on the mucosa can be significantly reduced. It may be reasonable to consider the main occluding areas [46] for each IARPD patient to determine the most optimum implant location.

Most studies showed that the load on the implant became larger in the implant location of the second molar area [17, 19, 22]. Other studies demonstrated that the bending moment of the implant [20] or peri-implant bone strain was larger in the implant location in the premolar location [18, 21]. Considering the stress in the entire jawbone, placing implants at the first molar region might be less stressful [23–25] and enhance balance [26]. However, the included FEA studies did not focus on the region of interest in the peri-implant bone for stress distribution. It is to be noted that the effect of bracing and retention of implant abutment can change denture behavior, affecting the load distribution in IARPD [22, 27]. Despite favorable clinical outcomes of the implants in IARPD [3, 5], there may be some concerns about peri-implant bone resorption; therefore, researchers should consider the burden on the implant in IARPD. On the other hand, defining an appropriate load distribution is difficult. The risk of mechanical or biological complications is thought to increase if the load is concentrated on any one supporting element in IARPD. Therefore, appropriate load distribution can be considered a state in which stress is not concentrated on any one supporting element.

Although the experimental studies included in this review reported the absolute values of load or mechanical stress on the supporting elements, they used them to assess the experimental conditions in each study. Thus, comparing the absolute values among the different studies was less meaningful. In addition, the effect of implant location on load distribution differed across studies, which may be attributed to the heterogeneity of the methodology used in these studies. In particular, model setting, loading condition, load or stress measuring methods, or assessment places were different. Due to the above limitations, the results were not analyzed statistically in this review. In addition, simulation studies warrant verifying the validity of simulation results with actual clinical data [47]. Although this review included the studies with IARPDs for three or more teeth-free-end missing, patients with two teeth-free-end missing also visit the dental clinic. Actually, one study included the case of two teeth free-end missing for both the model experiment and FEA and investigated the mechanical stress on the abutment tooth and implant of IARPD [48]. A shortened dental arch (no prosthesis or only implant-supported fixed prosthesis at the first molar) or a fixed prosthesis with two implants may be clinically adopted rather than the IARPD in such cases, but it is necessary to investigate IARPD for two missing teeth in the future.

Summarizing the studies comparing three implant locations in IARPD for mandibular free-end missing: the first or second premolar, first molar, and second molar areas, the effect of implant location differed among the studies due to the differences in the measurement method, such as the load measurement method or position, and loading conditions.

Overall, clinical suggestions can be provided for each implant position in the case of one implant-assisted removable partial denture in mandibular free-end missing.

*Premolar region* The condition of the peri-implant bone should be evaluated carefully, because the lateral load on the implant can be relatively high due to the rotational movement of the denture with the implant as a fulcrum. It is recommended when the abutment tooth is periodontally compromised and an implant in the premolar region would reduce the forces on the abutment tooth.

*First molar region* Considering the balance of load distribution to all the support elements of the IARPD, implant placement here may offer a greater favorable distribution and dissipation of load and stress among the various supporting elements.

*Second molar region* The load on the mucosa under the denture base may be reduced. The condition of the abutment tooth should be considered, and equal load distribution to the remaining teeth might be essential to prevent load concentration on the abutment tooth. It is recommended when periodontal conditions of the abutment tooth are stable.

## Conclusions

Within the limitations, this review of in vitro model experiments and FEA studies demonstrated the effects of implant location on the load distribution in IARPD. The implant location in IARPD can affect load distribution to the supporting elements, such as the abutment tooth, implant, and mucosa under the denture base.

### Supplementary Information

**Additional file 1.** **Table S1.** Reasons for exclusion of articles.

## Data Availability

The data sets generated and analyzed during the current study are available from the corresponding author upon reasonable request.

## Abbreviations

IARPD: Implant-assisted removable partial denture
CRPD: Conventional removable partial denture
2-D: 2-Dimensional
3-D: 3-Dimensional
FEA: Finite-element analysis
PDL: Periodontal ligament
RPA: Rest, proximal plate, and Akers clasps
RPI: Rest, proximal plate, and I-bar
CT: Computed tomography
