# Supplementary material for: Impact of implant location on load distribution of implant-assisted removable partial dentures: a review of in vitro model and finite-element analysis studies
Source: Int J Implant Dent. 2023 Sep 19;9:31. doi: 10.1186/s40729-023-00500-3 (PMC10509086; doi:10.1186/s40729-023-00500-3)
Supplement: Supplementary file 1 — Additional file 1. Table S1. Reasons for exclusion of articles. [file 40729_2023_500_MOESM1_ESM.pdf]

Exclusion Table

	Study (author/year)	Bibliographic reference	Reason for exclusion
1	Eom JW 2017	Ju-Won Eom , Young-Jun Lim , Myung-Joo Kim , Ho-Beom Kwon. Three-dimensional finite element analysis of implant-assisted removable partial dentures. J Prosthet Dent. 2017 Jun;117(6):735-742. doi: 10.1016/j.prosdent.2016.09.021. Epub 2016 Dec 1.	Dose not compare the location of the implant. Compare the various pattern of denture system.
2	Nogawa T 2020	Toshifumi Nogawa, Masayasu Saito, Naomichi Murashima, Yoshiyuki Takayama, Atsuro Yokoyama. Influence of rigidity of retainers on dynamic behavior of implant-supported removable partial dentures. Int J Implant Dent. 2020 Oct 22;6(1):60. doi: 10.1186/s40729-020-00260-4.	Dose not compare the location of the implant. Compare three types of direct retainers of IARPD.
3	Shahmiri R 2017	Reza Shahmiri, Raj Das. Finite element analysis of implant-assisted removable partial dentures: Framework design considerations. J Prosthet Dent. 2017 Aug;118(2):177-186. doi:10.1016/j.prosdent.2016.10.032. Epub 2017 Jan 12.	Dose not compare the location of the implant. Compare the stress and deformation patterns of the denture structure.
4	Shahmiri R 2013	Reza Shahmiri, John M Aarts, Vincent Bennani, Momen A Atieh, Michael V Swain. Finite element analysis of an implant-assisted removable partial denture. J Prosthodont. 2013 Oct;22(7):550-555. doi: 10.1111/jopr.12031. Epub 2013 Apr 1.	Dose not compare the location of the implant. Analyze deformation in the IARPD and elastic strain in the metal framework.
5	Kono K 2014	Kentaro Kono , Daisuke Kurihara , Yasunori Suzuki , Chikahiro Ohkubo .Pressure distribution of implant-supported removable partial dentures with stress-breaking attachments.J Prosthodont Res. 2014 Apr;58(2):115-20. doi: 10.1016/j.jpor.2014.01.002. Epub 2014 Mar 11.	Dose not compare the location of the implant. Compare the attachment system on the implant.
6	Shahmiri R 2014	Reza Shahmiri, Raj Das, John M Aarts, Vincent Bennani. Finite element analysis of an implant-assisted removable partial denture during bilateral loading: occlusal rests position. J Prosthet Dent. 2014 Nov;112(5):1126-33. doi: 10.1016/j.prosdent.2014.04.023. Epub 2014 Jun 18.	Dose not compare the location of the implant. Compare the stress and deformation patterns of the denture structure.
7	Gharehchahi J 2013	Jafar Gharehchahi, Nafiseh Asadzadeh, Amirtaher Mirmortazavi, Mohammad Taghi Shakeri. Maximum dislodging forces of mandibular implant-assisted removable partial dentures: in vitro assessment. J Prosthodont. 2013 Oct;22(7):543-549. doi: 10.1111/jopr.12048. Epub 2013 Apr 1.	Dose not compare the location of the implant. Compare maximum dislodging forces of IARPD.
8	Shahmiri R 2013	Reza Shahmiri, John M Aarts, Vincent Bennani, Raj Das, Michael V Swain. Strain Distribution in a Kennedy Class I Implant Assisted Removable Partial Denture under Various Loading Conditions. Int J Dent. 2013;2013:351279. doi: 10.1155/2013/351279. Epub 2013 Apr 30.	Dose not compare the location of the implant. Investigate the strain to the IARPD.
9	de Freitas Santos CM 2011	Ciandrus Moraes de Freitas Santos, Eduardo Piza Pellizzer, Fellippo Ramos Verri, Sandra Lúcia Dantas de Moraes, Rosse Mary Falcón-Antenucci. Influence of implant inclination associated with mandibular class I removable partial denture. J Craniofac Surg. 2011 Mar;22(2):663-8. doi: 10.1097/SCS.0b013e318207458b.	Dose not compare the location of the implant. Compare the different inclinations of the placed implant.
10	Ohkubo C 2007	Ohkubo C, Kurihara D, Shimpo H, Suzuki Y, Kokubo Y, Hosoi T. Effect of implant support on distal extension removable partial dentures: in vitro assessment. J Oral Rehabil. 2007 Jan;34(1):52-6. doi: 10.1111/j.1365-2842.2006.01641.x.	Dose not compare the location of the implant. Investigate the stability of IARPD.
11	Mousa MA 2021	Mohammed A Mousa, Johari Yap Abdullah, Nafij B Jamayet, Mohamed I El-Anwar, Kiran Kumar Ganji, Mohammad Khursheed Alam, Adam Husein. Biomechanics in Removable Partial Dentures: A Literature Review of FEA-Based Studies.Biomed Res Int. 2021 Aug 26;2021:5699962. doi: 10.1155/2021/5699962. eCollection 2021.	Review article
12	Archangelo CM 2012	Carlos Marcelo Archangelo, Eduardo Passos Rocha, João Antônio Pereira, Manoel Martin Junior, Rodolfo Bruniera Anchieta, Amilcar Chagas Freitas Júnior. Periodontal ligament influence on the stress distribution in a removable partial denture supported by implant: a finite element analysis. J Appl Oral Sci. 2012 May-Jun;20(3):362-8. doi: 10.1590/s1678-77572012000300012.	Dose not compare the location of the implant. Assess the influence of non-homogenous PDL on the stress distribution.
13	Arita S 2021	Shuhei Arita, Tomoya Gonda, Hitomi Togawa, Yoshinobu Maeda, Kazunori Ikebe. Influence of mandibular distal extension implant-supported removable partial dentures on the force exerted on maxillary anterior teeth. J Prosthodont Res. 2021 Oct 15;65(4):541-545. doi: 10.2186/jpr.JPR_D_20_00077. Epub 2021 Apr 23.	Dose not compare the location of the implant. Measure the load exerted on the maxillary anterior teeth.
14	Mozayek RS 2016	Rami Shurbaji Mozayek, Mohammad Yamen Shurbaji Mozayek, Mirza Allaf, Mohammad Bassam Abouharb. The effectiveness of adding a supporting implant in stress distribution of long span fixed partial denture (three-dimensional finite element analysis). J Indian Prosthodont Soc. 2016 Jul-Sep;16(3):259-63. doi: 10.4103/0972-4052.176533.	Not investigated for IARPD
15	Verri FR 2011	Fellippo Ramos Verri, Eduardo Piza Pellizzer, João Antônio Pereira, Paulo Renato Junqueira Zuim, Joel Ferreira Santiago Júnior. Evaluation of bone insertion level of support teeth in class I mandibular removable partial denture associated with an osseointegrated implant: a study using finite element analysis. Implant Dent. 2011 Jun;20(3):192-201. doi: 10.1097/ID.0b013e3182166927.	Dose not compare the location of the implant. Evaluate the different bone level of abutment tooth.
16	Sato M 2013	Maki Sato , Yasunori Suzuki, Daisuke Kurihara, Hidemasa Shimpo, Chikahiro Ohkubo. Effect of implant support on mandibular distal extension removable partial dentures: relationship between denture supporting area and stress distribution. J Prosthodont Res. 2013 Apr;57(2):109-12. doi: 10.1016/j.jpor.2013.01.002. Epub 2013 Apr 10.	Dose not compare the location of the implant. Compare the stability of IARPD and conventional RPD.
17	Fayaz A 2015	Amir Fayaz, Alahyar Geramy, Yeganeh Memari, Zahra Rahmani. Effects of Length and Inclination of Implants on Terminal Abutment Teeth and Implants in Mandibular CL1 Removable Partial Denture Assessed by Three-Dimensional Finite Element Analysis. J Dent (Tehran). 2015 Oct;12(10):739-46.	Dose not compare the location of the implant. Assess the effects of length and inclination of implants on stress distribution in IARPD.
18	Mahshid M 2014	Minoo Mahshid, Allahyar Geramy, Massoud Ejlali, Majid Sedaghat Monfared, Shirin Rezvani Habib Abadi. Effect of the number of implants on stress distribution of anterior implant-supported fixed prostheses combined with a removable partial denture: a finite element analysis. J Dent (Tehran). 2014 May;11(3):335-42. Epub 2014 May 31.	Not investigated for IARPD
19	Cunha LDAP 2011	Lígia Del' Arco Pignatta Cunha, Eduardo Piza Pellizzer, Fellippo Ramos Verri, Rosse Mary Falcón-Antenucci, Marcelo Coelho Goiato. Influence of ridge inclination and implant localization on the association of mandibular Kennedy class I removable partial denture. J Craniofac Surg. 2011 May;22(3):871-5. doi: 10.1097/SCS.0b013e31820f7d6a.	Dose not compare the effect of location of the implant on load distribution quantitatively.
20	Elsyad MA 2017	Moustafa Abdou ELsyad, Abdelbaset Omar Omran, Mohammed Mohammed Fouad. Strains Around Abutment Teeth with Different Attachments Used for Implant-Assisted Distal Extension Partial Overdentures: An In Vitro Study.J Prosthodont. 2017 Jan;26(1):42-47. doi: 10.1111/jopr.12370. Epub 2015 Sep 29.	Dose not compare the location of the implant. Compare the attachment system on implant in IARPD.
21	Verri FR 2007	Fellippo Ramos Verri, Eduardo Piza Pellizzer, Eduardo Passos Rocha, João Antônio Pereira. Influence of length and diameter of implants associated with distal extension removable partial dentures. Implant Dent. 2007 Sep;16(3):270-80. doi: 10.1097/ID.0b013e31805007aa.	Dose not compare the location of the implant. Compare the length and diameter of implants in IARPD.
22	Hirata K 2017	Kiyotaka Hirata, Toshihito Takahashi, Akiko Tomita, Tomoya Gonda, Yoshinobu Maeda. Influence of Abutment Angle on Implant Strain When Supporting a Distal Extension Removable Partial Dental Prosthesis: An In Vitro Study.Int J Prosthodont. 2017 Jan/Feb;30(1):51-53. doi: 10.11607/ijp.5010.	Dose not compare the location of the implant. Compare the abutment angle on implant in IARPD.
23	Xiao W 2016	Wei Xiao, Zhiyong Li, Shiqian Shen, Shaowu Chen, Sulin Chen, Jiawei Wang. Influence of connection type on the biomechanical behavior of distal extension mandibular removable partial dentures supported by implants and natural teeth.Comput Methods Biomech Biomed Engin. 2016 Feb;19(3):240-247. doi: 10.1080/10255842.2015.1009450. Epub 2015 Feb 18.	Dose not compare the location of the implant. Compare the connection type between implant and IARPD.
24	Koodaryan R 2022	Koodaryan, R, Yasamineh N, Hafezeqoran A, Moharrer SM, Noorazar SG. Determination of strain around distal implants as abutments for partial and fixed implant-supported prostheses with posterior cantilever. Journal of Advanced Pharmaceutical Technology and Research. 13.2.100-105. 2022. 10.4103/japtr.japtr_160_21	Not investigated for IARPD
25	Rungsiyakull C 2022	Rungsiyakull C, Rungsiyakull P, Suttiat K, Duangrattanaprathip N. Stress Distribution Pattern in Mini Dental Implant-Assisted RPD with Different Clasp Designs: 3D Finite Element Analysis. International Journal of Dentistry. 2416888. 2022. 10.1155/2022/2416888	Dose not compare the location of the implant. Compare the clasp design of direct retaner of IARPD.
26	Messias A 2021	Messias A, Neto MA, Amaro AM, Lopes VM, Nicolau P. Mechanical evaluation of implant-assisted removable partial dentures in kennedy class i patients: Finite element design considerations. Applied Sciences (Switzerland). 11.2.659.1-18. 10.3390/app11020659. 2021	Dose not compare the location of the implant. Compare the stress distribution of the frame of IARPD.
27	Tribst JPM 2020	Tribst JPM, de Araújo RM, Ramanzine NP, Santos NR, de Oliveira Dal Piva AM, Borges ALS, da Silva JMF. Mechanical behavior of implant assisted removable partial denture for Kennedy class II. Journal of Clinical and Experimental Dentistry. 12.16533. e38-e45. 2020. 10.4317/MEDORAL.56533	IARPD for 2 teeth-free-end missing was targeted.
28	Bilhan SA 2019	Bilhan SA, Geckili O, Cilingir A, Bozdag E, Bilhan H. Evaluation of two interforaminal implants and implant-assisted removable dentures on stress distribution: An in vitro study. Journal of the Korean Association of Oral and Maxillofacial Surgeons. 45.4.199-206. 2019. 10.5125/jkaoms.2019.45.4.199	Not investigated for IARPD
29	Jensen C 2017	Jensen C, Speksnijder CM, Raghoebar GM, Kerdijk W, Meijer HJA, Cune MS. Implant-supported mandibular removable partial dentures: Functional, clinical and radiographical parameters in relation to implant position. Clinical Implant Dentistry and Related Research. 19-3.432-439. 2017. 10.1111/cid.12484	Clinical study
30	Shahmiri R 2016	Shahmiri R, Das R. Finite element analysis of implant-assisted removable partial denture attachment with different matrix designs during bilateral loading. International Journal of Oral and Maxillofacial Implants. 31.5.e116-e127. 2016. 10.11607/jomi.4400	Dose not compare the location of the implant. Investigate the effect of different matrix designs on resilient attachment on IARPD.
31	Hirata K 2016	Hirata K, Takahashi T, Tomita A, Gonda T, Maeda Y. Loading variables on implant-supported distal-extension removable partial dentures: An in vitro pilot study. International Journal of Prosthodontics. 29.1.17-19. 2016. 10.11607/ijp.4411	Dose not compare the location of the implant. Compare the effect of inclination of implant in IARPD.
32	Hirata K 2015	Hirata K, Takahashi T, Tomita A, Gonda T, Maeda Y. The influence of loading variables on implant strain when supporting distal-extension removable prostheses: An in vitro study. International Journal of Prosthodontics. 28.5.484-486. 2015. 10.11607/ijp.4208	Dose not compare the location of the implant. Compare the occlusal force direction to IARPD.
33	Gharehchahi J 2013	Gharehchahi J, Asadzadeh N, Mirmortazavi A, Shakeri MT. Maximum dislodging forces of mandibular implant-assisted removable partial dentures: In vitro assessment. Journal of Prosthodontics. 22.7.543-549, 2013. 10.1111/jopr.12048	Dose not compare the location of the implant. Compare the maximum dislodging forces of IARPD.
34	Saad AN 2012	Saad AN, Eid HI, Elsayed MEM, El-Ragiand A, Zawahry MME. Stresses around the abutment teeth and implants supporting an implant supported removable partial denture. Journal of Applied Sciences Research. 8.8.4308-4315.	Dose not compare the location of the implant. Compare the effect of inclination of implant in IARPD.
35	Santos CMDF 2011	Santos CMDF, Pellizzer EP, Verri FR, De Moraes SLD, Falcón-Antenucci RM. Influence of implant inclination associated with mandibular class I removable partial denture. Journal of Craniofacial Surgery. 22.2.663-668. 2011. 10.1097/SCS.0b013e318207458b	Dose not compare the location of the implant. Compare the effect of attachement type on implant in IARPD.
